# Large primary retroperitoneal mucinous cystadenoma: laparoscopic management and comprehensive literature review

**DOI:** 10.1093/jscr/rjag546

**Published:** 2026-09-09

**Authors:** Athanasios Kofinas, Kalliopi E Stavrati, Stella Vasileiadou, Konstantina E Karakasi, Kalliopi Gianna, Alexandra Marneri, Christina Mouratidou, Efstathios T Pavlidis, Nikolaos Antoniadis, Georgios Katsanos, Georgios Tsoulfas, Theodoros E Pavlidis

**Affiliations:** Department of Transplantation Surgery, Center for Research and Innovation in Solid Organ Transplantation, Aristotle University of Thessaloniki, School of Medicine, Konstantinoupoleos 49, Thessaloniki 54642, Greece; The Second Department of Propaedeutic Surgery, Hippokration General Hospital, School of Medicine, Aristotle University of Thessaloniki, Konstantinoupoleos 49, Thessaloniki 54642, Greece; Department of Transplantation Surgery, Center for Research and Innovation in Solid Organ Transplantation, Aristotle University of Thessaloniki, School of Medicine, Konstantinoupoleos 49, Thessaloniki 54642, Greece; Department of Transplantation Surgery, Center for Research and Innovation in Solid Organ Transplantation, Aristotle University of Thessaloniki, School of Medicine, Konstantinoupoleos 49, Thessaloniki 54642, Greece; Department of Pathology, Ippokrateio General Hospital of Thessaloniki, Konstantinoupoleos 49, Thessaloniki, Greece; Intensive Care Unit, Hippokration General Hospital, Konstantinoupoleos 49, Thessaloniki 54642, Greece; Intensive Care Unit, Hippokration General Hospital, Konstantinoupoleos 49, Thessaloniki 54642, Greece; The Second Department of Propaedeutic Surgery, Hippokration General Hospital, School of Medicine, Aristotle University of Thessaloniki, Konstantinoupoleos 49, Thessaloniki 54642, Greece; Department of Transplantation Surgery, Center for Research and Innovation in Solid Organ Transplantation, Aristotle University of Thessaloniki, School of Medicine, Konstantinoupoleos 49, Thessaloniki 54642, Greece; Department of Transplantation Surgery, Center for Research and Innovation in Solid Organ Transplantation, Aristotle University of Thessaloniki, School of Medicine, Konstantinoupoleos 49, Thessaloniki 54642, Greece; Department of Transplantation Surgery, Center for Research and Innovation in Solid Organ Transplantation, Aristotle University of Thessaloniki, School of Medicine, Konstantinoupoleos 49, Thessaloniki 54642, Greece; The Second Department of Propaedeutic Surgery, Hippokration General Hospital, School of Medicine, Aristotle University of Thessaloniki, Konstantinoupoleos 49, Thessaloniki 54642, Greece

**Keywords:** mucinous cystadenoma, retroperitoneal mass, laparoscopic surgery

## Abstract

Primary retroperitoneal mucinous cystadenoma (PRMC) is a rare cystic tumor with unclear etiology and nonspecific clinical and radiological features, making preoperative diagnosis difficult. Due to its rarity, the optimal surgical management remains uncertain. We report a 60-year-old woman presenting with diffuse, colicky abdominal pain. Computed tomography and magnetic resonance imaging revealed a large, well-defined cystic mass in the right retroperitoneum measuring 12 × 9 × 10 cm, without evidence of local invasion. The patient underwent successful laparoscopic excision. Histopathological examination demonstrated a unilocular cyst lined by non-atypical mucinous epithelium without stromal invasion, confirming PRMC. The postoperative course was uneventful, and no recurrence was observed during follow-up. PRMC should be included in the differential diagnosis of retroperitoneal cystic masses. Histopathological evaluation is essential for definitive diagnosis. Complete surgical excision remains the treatment of choice, while laparoscopy may represent a safe and effective approach in selected patients when careful operative technique is applied.

## Introduction

Primary mucinous cystadenomas are uncommon cystic lesions located in the retroperitoneum, and their etiology remains uncertain due to the scarcity of reported cases. Histologically, they resemble ovarian mucinous cystic neoplasms but are distinct entities unrelated to ovarian pathology [1]. Typically, these lesions are discovered incidentally or present with nonspecific symptoms such as diffuse pain or flatulence. Diagnosis primarily relies on histopathological examination, as radiological features lack specificity. Surgical intervention, involving complete excision of the cyst, is the cornerstone of treatment. Although laparotomy has traditionally been the preferred surgical approach, minimally invasive techniques have been increasingly reported in selected cases. We present a case of successful laparoscopic management of a large primary retroperitoneal mucinous cystadenoma (PRMC) and provide a review of the previously reported cases in the literature. This study presents a case of successful laparoscopic surgery for PRMC and provides a comprehensive review of literature.

## Case report

**Figure 1:**
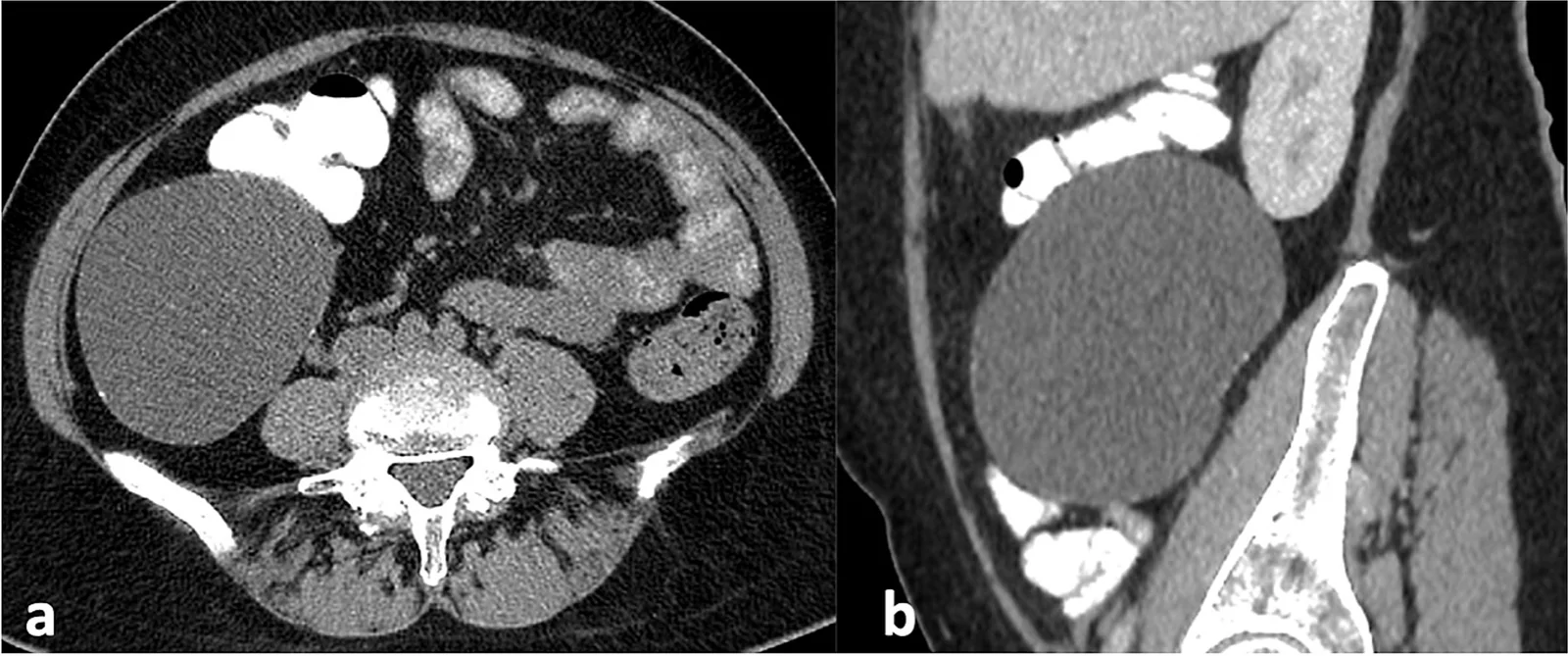
(a, b) Multidetector CT : axial (a) and sagittal (b) images before and after i.v. contrast, respectively. There is a large cystic mass situated in the right retroperitoneum inferior to the right kidney and behind the ascending colon. Its capsule is thin with tiny scattered calcifications. Images after i.v. contrast medium administration reveal mild peripheral enhancement.

**Figure 2:**
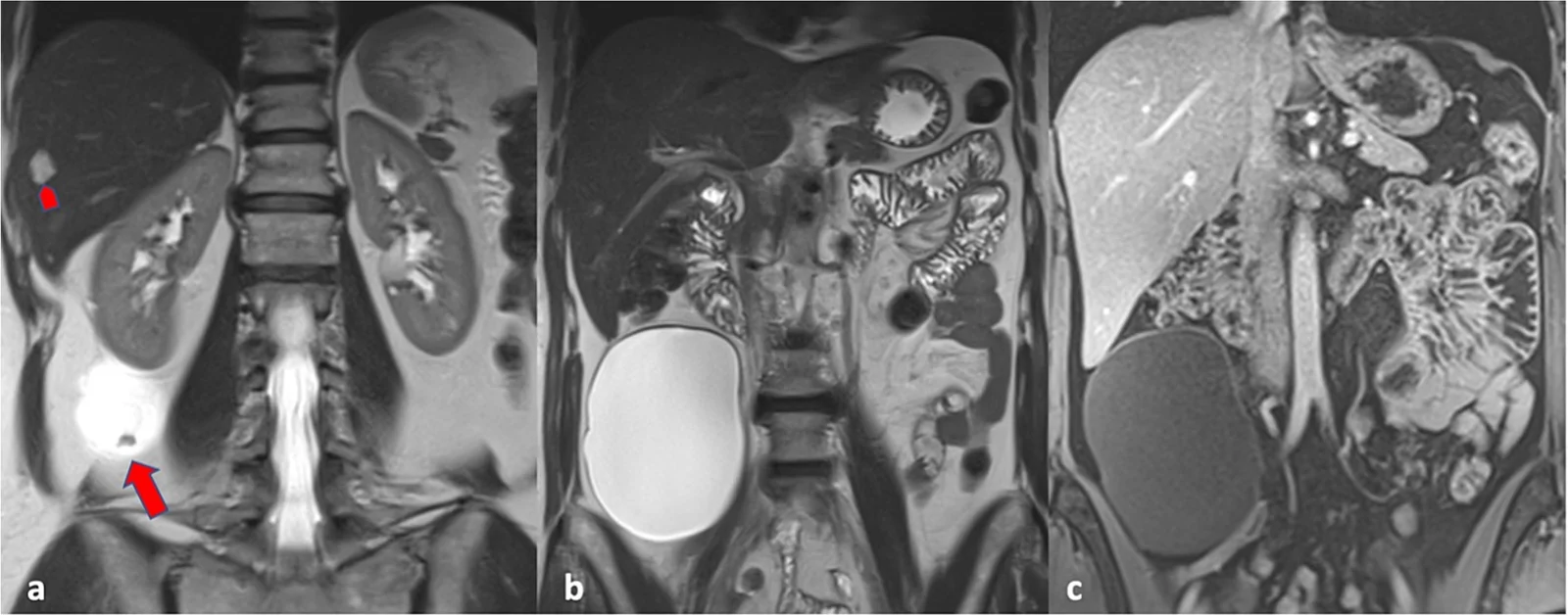
(a–c) MRI: coronal T2 WI (a, b) and T1 WI after i.v. contrast administration. (c) The large cystic mass is hyperintense and hypointense on T2 WI and T1 WI, respectively. Its wall is thin and well-defined without evidence of internal septations or papillary projections. A small peripheral hypointense calcified nodule is noted (big arrow). There is only mild peripheral enhancement after i.v. contrast administration. A small hemangioma is demonstrated at the right liver lobe (small arrow).

A 60-year-old woman presented at the Emergency Department with diffuse abdominal colicky pain. No other concomitant symptoms were reported. On physical examination of the abdomen, distension and mild tenderness were evident. Her medical history included surgical repair of rectocele and cystocele 10 years earlier. Computed tomography (CT) scan revealed a large cystic mass located in the right retroperitoneum inferior to the right kidney and behind the ascending colon measuring 12 × 9 × 10 cm, with thin capsule and tiny scattered calcifications (Fig. 1). Routine laboratory tests and serum tumor markers (carcinoembryonic antigen [CEA], CA-125, CA 19-9, aFP) were within normal limits. Magnetic resonance imaging (MRI) demonstrated a well-defined cystic lesion that was hyperintense on T2-weighted images and hypointense on T1-weighted images, without internal septations or papillary projections (Fig. 2).

**Figure 3:**
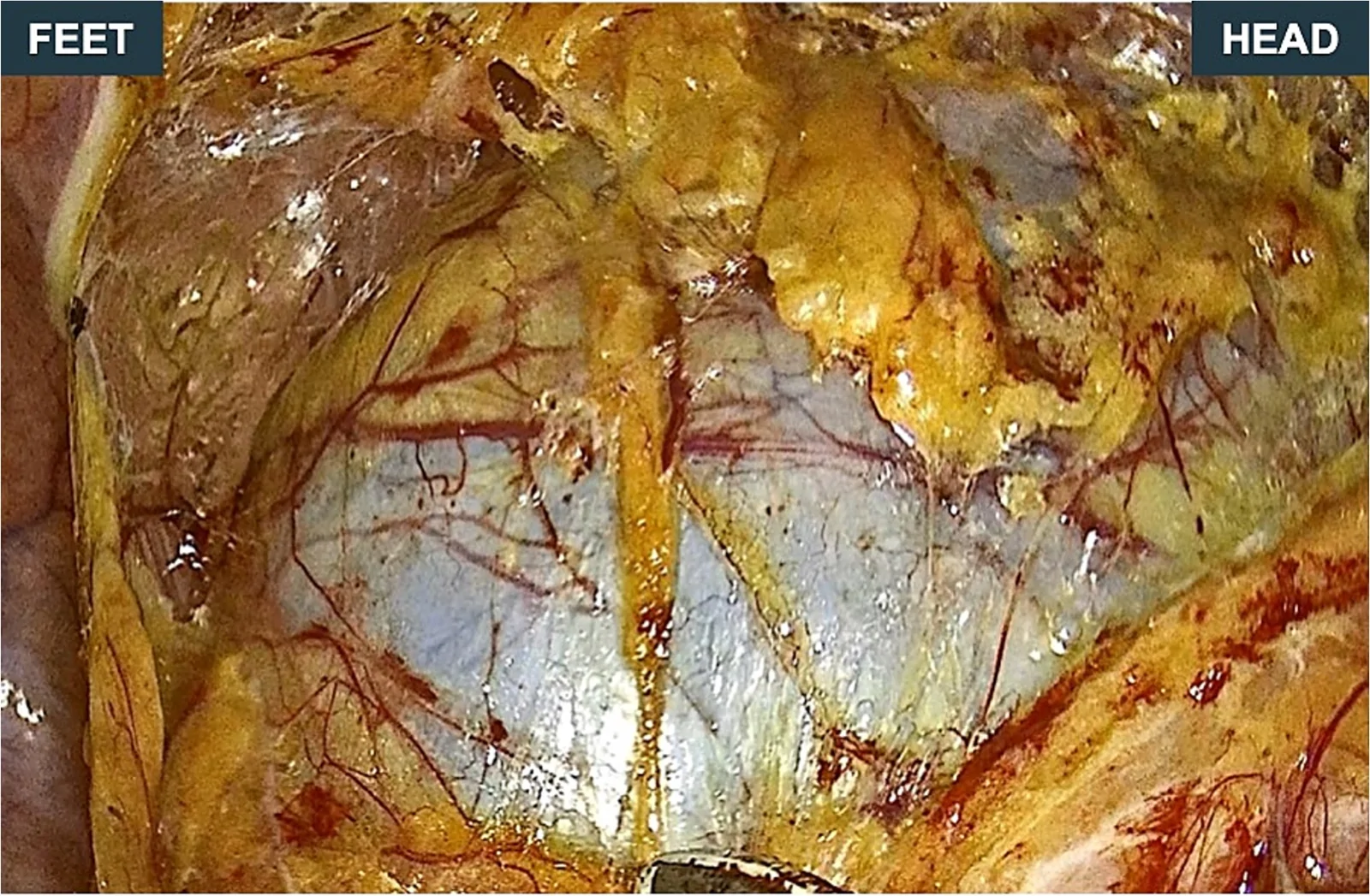
Laparoscopic view of the cyst.

**Figure 4:**
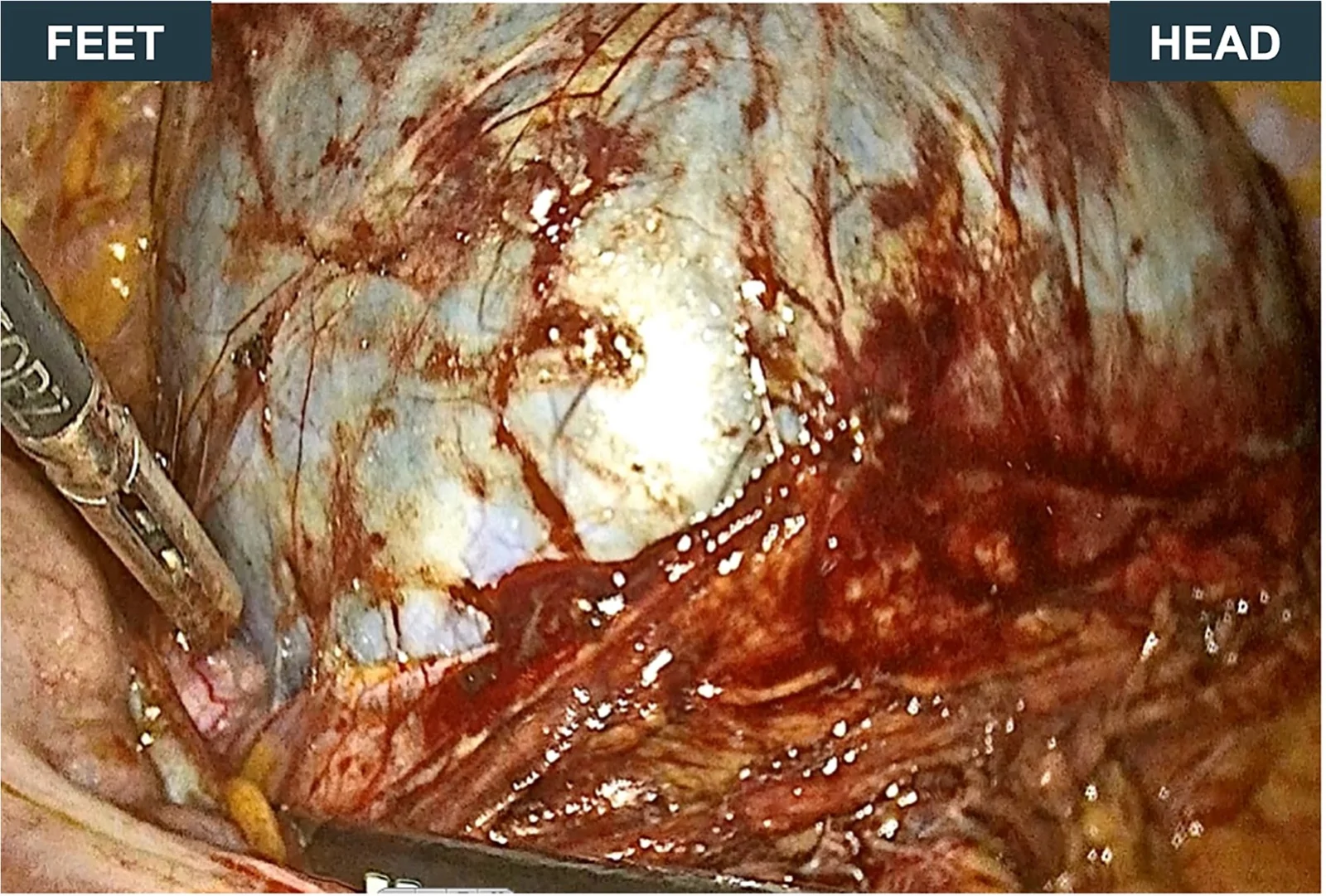
Careful separation of the cyst from the mesocolon.

**Figure 5:**
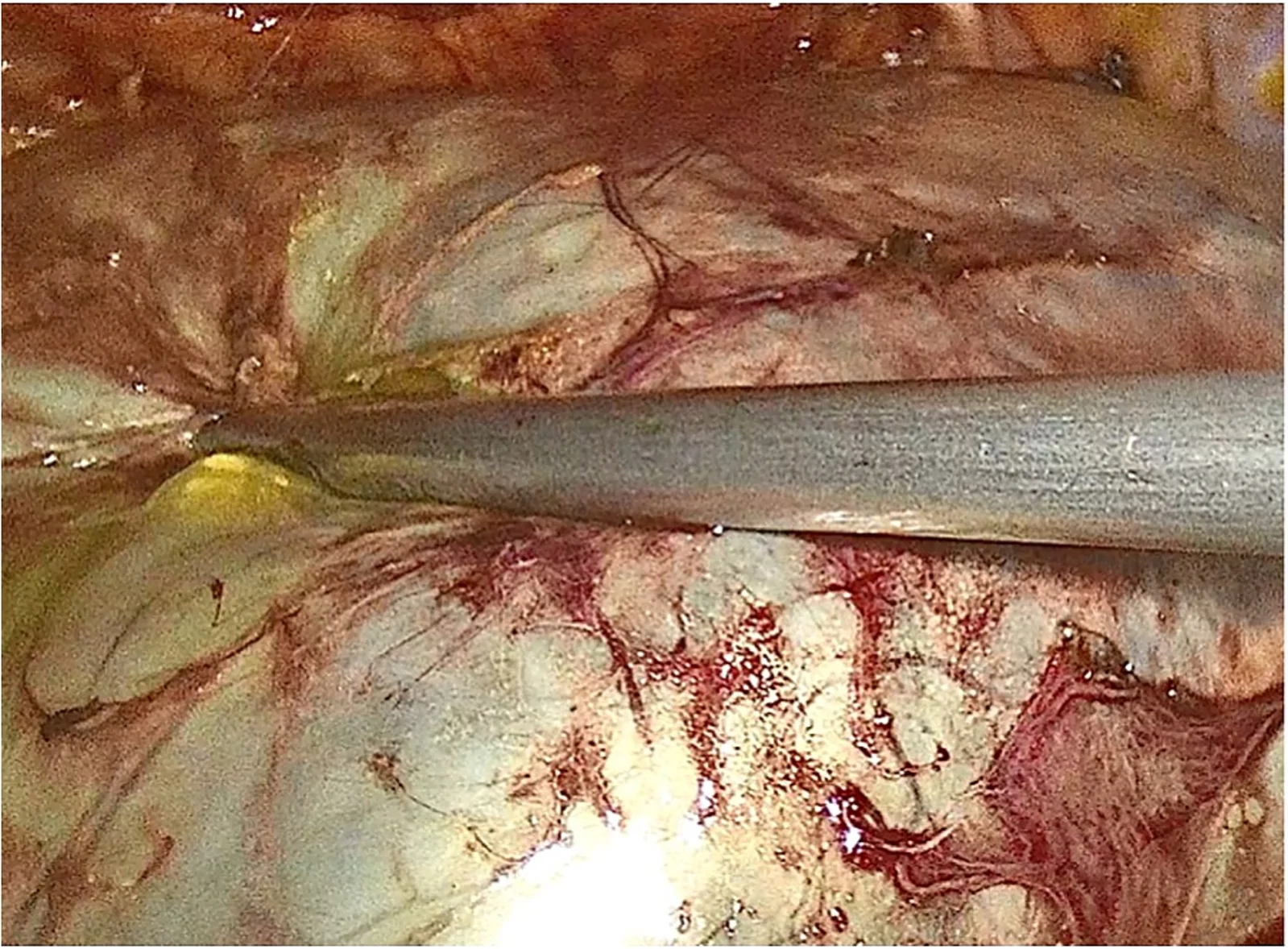
Drainage of the cystic fluid.

**Figure 6:**
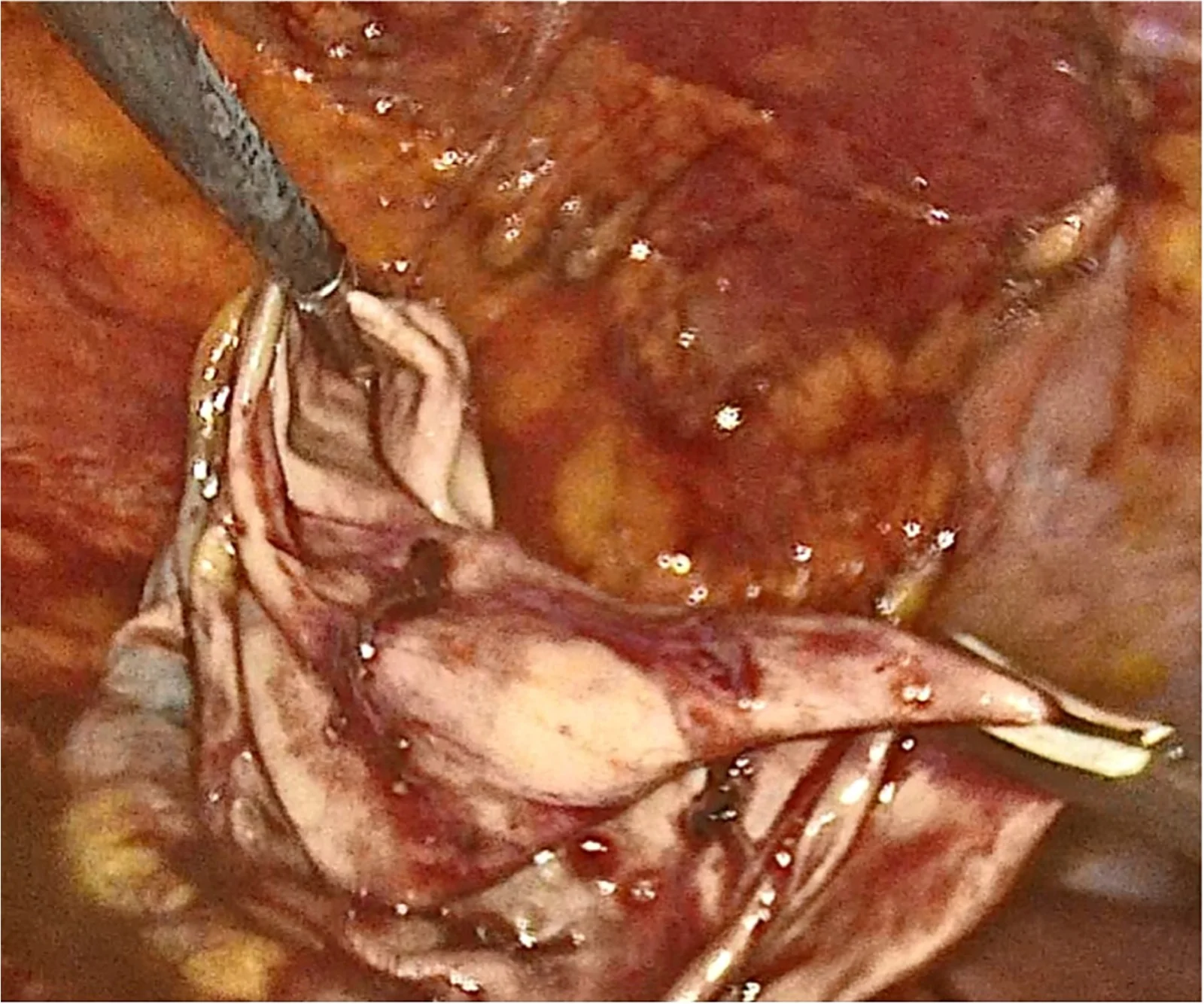
Cyst being placed in a collection bag.

**Figure 7:**
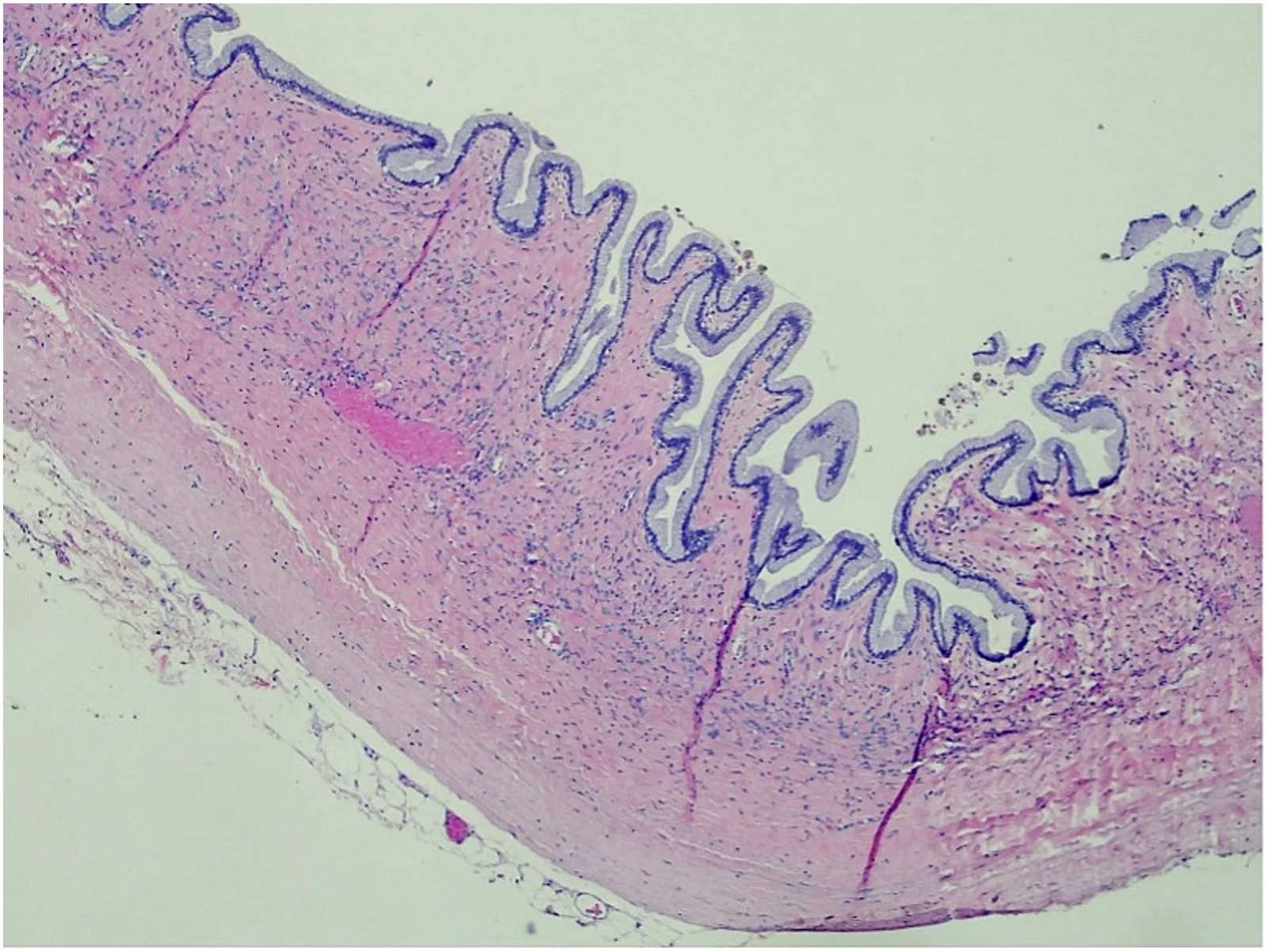
Cystic wall of a retroperitoneal neoplasm (HE × 40).

**Figure 8:**
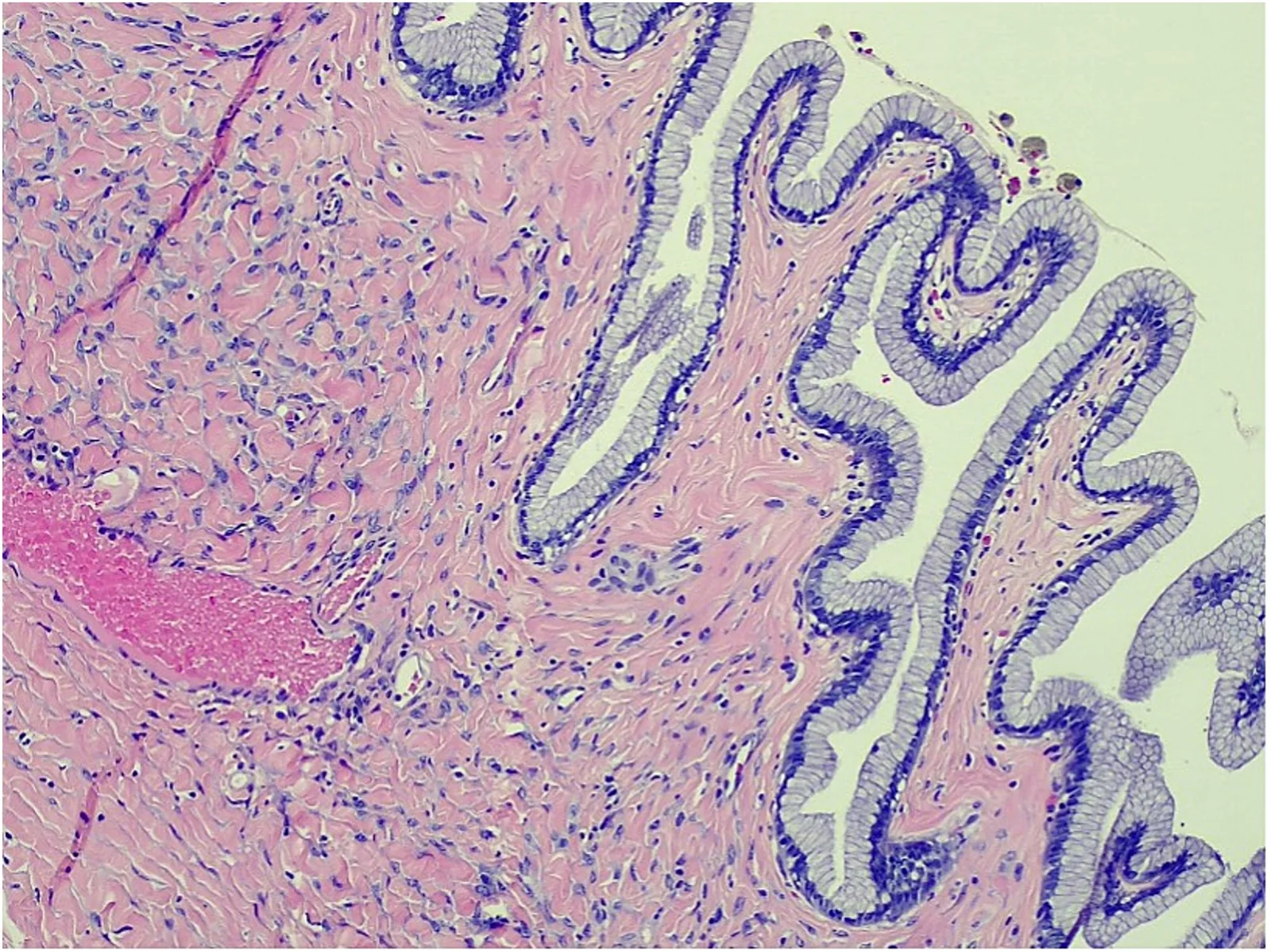
The cystic wall is lined by mucinous epithelium.

**Figure 9:**
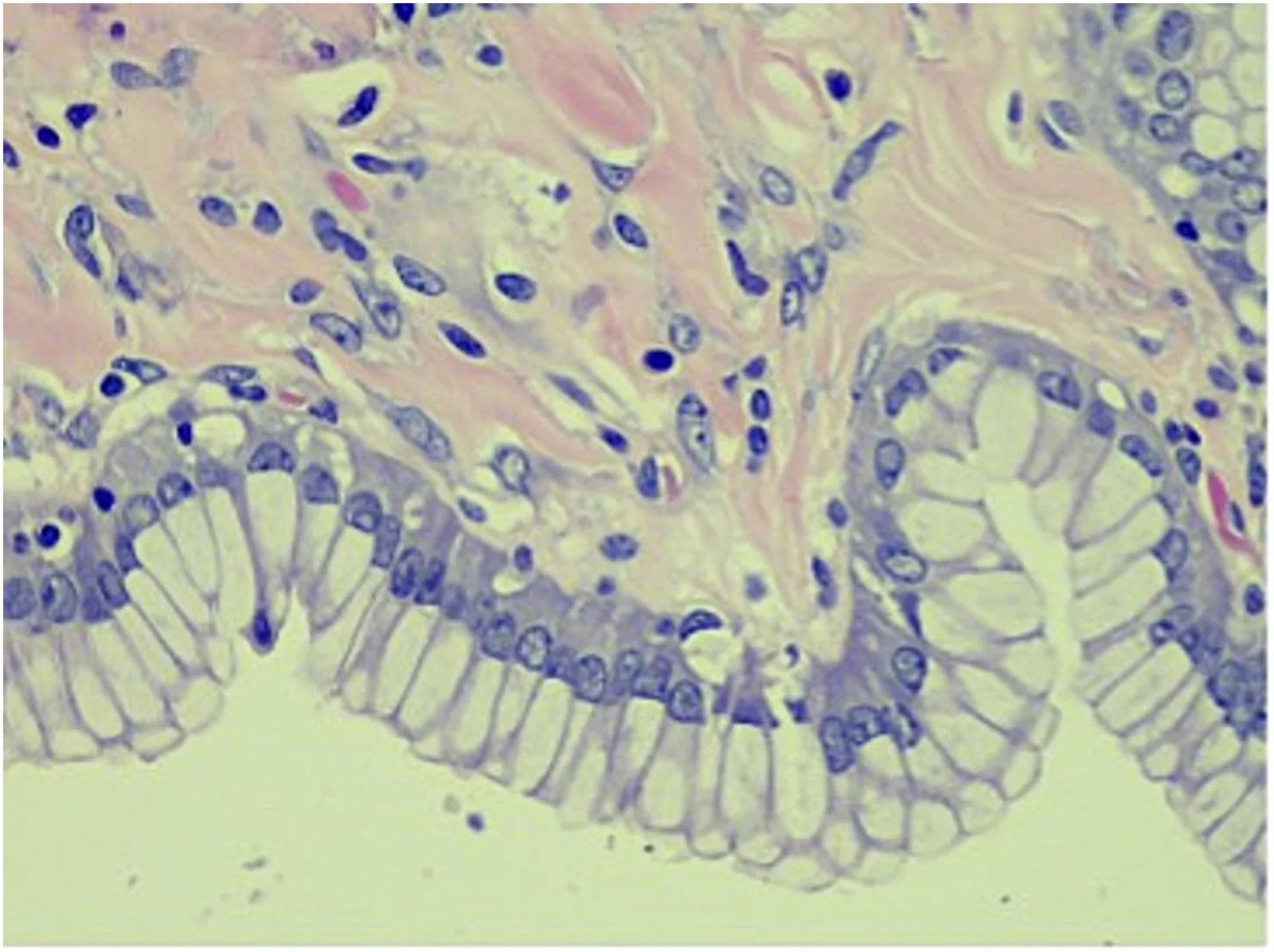
The epithelial cells exhibit basally located small, round nuclei, lacking cytological atypia (HE ×400).

In the present case, the decision to proceed laparoscopically, despite the considerable size of the mass, was based on the absence of radiological findings suspicious for malignancy. Under general anesthesia with endotracheal intubation, the patient was placed in a supine position. A 12-mm port was inserted through a small left paraumbilical incision after establishment of pneumoperitoneum, followed by placement of an 11-mm and a 5-mm port in the left lateral abdominal wall. Careful dissection and controlled decompression enabled safe mobilization and retrieval of the specimen while minimizing the risk of inadvertent rupture (Figs 3–5). Additionally, a specimen retrieval bag was used for extraction in order to prevent potential cell dissemination in the abdominal wall. The cytology revealed many macrophages, sometimes with endoprotoplasmic hemosiderin granules and a few cellular rachis and no evidence of malignancy. The cyst was removed through the 11 mm port after it was placed in a collection bag (Fig. 6). The pathological report describes histological picture compatible with mucinous cystadenoma (Fig. 7), on the walls of which microcalcifications are observed. The cystic wall is lined by mucinous epithelium (Fig. 8). The epithelial cells exhibit basally located small, round nuclei, lacking cytological atypia (HE × 400) (Fig. 9). Multiple sections were examined and no atypia or stromal invasion was presented. The postoperative course was uneventful, and the patient was discharged on the second postoperative day. Follow-up MRI examinations at three and six months demonstrated no evidence of recurrence.

## Discussion

PRMCs represent rare cystic lesions located in the retroperitoneal region, initially documented by Bassini in 1889 [2]. Histologically resembling ovarian mucinous cystic neoplasms, these cysts can emerge independently within the retroperitoneal space, dissociated from ovarian involvement.

Clinical presentation is usually nonspecific and mainly related to the size of the lesion and compression of adjacent organs. The majority of reported cases involve female patients, while only a limited number of male cases have been described in the literature. The exact pathogenesis remains unclear. Proposed hypotheses include origin from ectopic ovarian tissue and mucinous metaplasia of invaginated mesothelial cells within the retroperitoneum [3–6].

A comprehensive review of existing literature categorizes retroperitoneal mucinous tumors into mucinous cystadenomas, mucinous borderline tumors of low malignant potential, and mucinous cystadenocarcinomas, with benign lesions demonstrating a low propensity for malignant transformation [7].

The documented cases of primary retroperitoneal cystadenomas in published literature are scarce, numbering less than sixty cases and are presented at Table 1 [8, 9]. Among the 53 reported cases, 49 occurred in women and 4 in men. Patient age ranged from 14 to 85 years. Laparotomy was the most frequently reported approach, whereas minimally invasive techniques have been increasingly adopted in recent years.

**Table 1:** Documented cases of primary retroperitoneal cystadenomas

	Author	Year	Sex	Age	Size (cm)	Approach
1	Pennell [13]	1989	Female	19	6 × 10	Laparotomy
2	Yunoki [14]	1998	Female	45	9 × 6	Laparotomy
3	Kehagias [15]	1999	Female	21	10 × 6	Laparotomy
4	Subramony [16]	2001	Female	25	30 × 25 × 10	Laparotomy
5	Balat [17]	2001	Female	44	Not reported	Laparotomy
6	Tamura [18]	2003	Female	14	13 × 9 × 15	Laparoscopy converted to open
7	Erdemoglu [19]	2003	Female	39	18 × 13 × 7	Laparotomy
8	Arribas [20]	2004	Female	39	10 × 9 × 5	Exploratory laparoscopy
9	Min [21]	2004	Female	38	10 × 7.5 × 5.5	Laparotomy
10	Isse [22]	2004	Female	18	11 × 8 × 7	Laparotomy
11	Isse [23]	2004	Female	85	21 × 14 × 8	Laparotomy
12	Lai [23]	2006	Male	52	Not reported	Not reported
13	Bakker [24]	2007	Female	45	20 × 11	Laparotomy
14	Prabhuraj [25]	2008	Male	45	27 × 15 × 16	Laparotomy
15	Yan [26]	2008	Female	29	20 × 14 × 6	Laparotomy
16	Tapper [27]	2009	Female	37	30 × 12 × 11	Laparotomy
17	Abedalthagafi [28]	2009	Female	44	11 × 7	Laparoscopy
18	Rifki Jai [1]	2009	Female	43	32 × 20	Laparotomy
19	Roma [29]	2009	Female	40	7	Not reported
20	Roma [29]	2009	Female	36	9	Not reported
21	Papadopoulou [30]	2011	Female	24	14 × 13 × 7	Laparotomy
22	Fujita [31]	2011	Female	29	18 × 13 × 12	Laparoscopy
23	Cheng [32]	2012	Not reported	Not reported	Not reported	Not reported
24	Cheng [32]	2012	Not reported	Not reported	Not reported	Not reported
25	Demirel [33]	2012	Female	34	14 × 10 × 9	Laparotomy
26	Navin [34]	2012	Female	30	Not reported	Laparoscopy converted to open
27	Fujita [35]	2012	Male	71	25	Not reported
28	Mattei [36]	2013	Male	32	10	Laparoscopy
29	Paraskevakou [7]	2014	Female	23	10	Laparoscopy
30	Paraskevakou [7]	2014	Female	23	2.5	Laparoscopy
31	Nam YJ [37]	2014	Female	21	5.5 × 3.5	Laparotomy
32	Santo-Filho MA [38]	2014	Female	21	15 × 12.5 × 5.5	Laparotomy
33	Knezevic S [39]	2015	Female	60	12.3 × 10.8	Laparotomy
34	Lee SE [40]	2015	Female	31	6.5	Laparoscopy
35	Lee SY [9]	2016	Female	31	8.9 × 9.7 × 10	Laparoscopy
36	Dayan D [41]	2016	Female	36	15	Laparoscopy
37	Vicario FJ [42]	2016	Female	35	14 × 11 × 10.5	Laparotomy
38	Zevallos Quiroz JC [43]	2016	Female	20	16 × 12 × 6	Laparotomy
39	Nardi [5]	2017	Female	50	17.1 × 15.5 × 10.8	Laparotomy
40	Pesapane [8]	2018	Female	52	8 × 5 × 1	Laparoscopy
41	Koyama R [44]	2019	Female	41	5 × 2.2 × 3	Laparoscopy
42	Foula MS [45]	2019	Female	29	13 × 11	Laparoscopy
43	Koyama R [46]	2019	Female	39	2	?
44	Lung J [47]	2019	Female	22	8.0 × 8.4 × 9.4	Laparoscopy
45	Danen C [48]	2020	Female	19	5.8 × 3.9 × 5.8	Laparoscopy
46	Afzal Z [49]	2020	Female	32	17 × 7 × 12	Laparotomy
47	Frini [50]	2022	Female	31	11	Laparotomy
48	Ali Taherinezhad Ledari [51]	2022	Female	20	20 × 15	Laparotomy
49	Laham [52]	2023	Female	23	Not reported	Laparotomy
50	Lu [53]	2024	Female	25	7.2 × 5.6	Laparoscopy
51	Mudhher [54]	2024	Female	59	6.5 × 8.8	Robotic
52	Liu Y [55]	2025	Female	29	20 × 10 × 10	Laparotomy
53	Current Case	2026	Female	60	12 × 9 × 10	Laparoscopy

Preoperative diagnosis remains difficult because imaging findings are often nonspecific. CT and MRI are important for evaluating lesion size, wall characteristics, calcifications, internal septations, and possible invasion of adjacent structures [10]. Definitive diagnosis hinges upon histopathological analysis. In our case, the absence of solid components, papillary projections, or radiological evidence of invasion supported the decision to proceed with a minimally invasive approach.

Measurement of CEA levels in aspiration fluid may serve as an adjunctive diagnostic tool [11]. This incident was firstly reported by Motoyama *et al.* in 1993, who demonstrated elevated CEA levels in patients with retroperitoneal mucinous tumors [12].

Complete surgical excision remains the treatment of choice for PRMCs. Historically, most reported cases have been managed in the past through laparotomy. However, laparoscopic management has increasingly been reported in selected patients. In large cystic lesions, minimally invasive surgery may be technically challenging because of limited working space and the potential risk of cyst rupture.

In the present case, controlled decompression of the cyst was intentionally performed under direct visualization to facilitate safer manipulation and retrieval of the specimen using a specimen retrieval bag. Particular care was taken to avoid uncontrolled intraperitoneal spillage. We believe that laparoscopic management may represent a safe and effective option in selected patients when preoperative imaging does not suggest malignancy and meticulous operative technique is applied.

## Conclusion

In the case of PRMCs, complete surgical excision is the recommended management strategy. Complete surgical excision remains the treatment of choice and definitive diagnosis is established histopathologically. In selected patients, laparoscopic management may represent a safe and effective approach when careful operative technique is applied to minimize the risk of cyst rupture and intraperitoneal contamination.
